# The Effect of Different Phases of Synchrony on Pain Threshold in a Drumming Task

**DOI:** 10.3389/fpsyg.2017.01034

**Published:** 2017-06-22

**Authors:** Philip Sullivan, Mishka Blacker

**Affiliations:** Department of Kinesiology, Brock University, St. CatharinesON, Canada

**Keywords:** synchrony, pain threshold, drumming

## Abstract

Behavioral synchrony has been linked to endorphin activity (Cohen et al., 2010; Sullivan and Rickers, 2013; Sullivan et al., 2014; Tarr et al., 2015, 2016; Weinstein et al., 2016). This has been called the synchrony effect. Synchrony has two dominant phases of movement; in-phase and anti-phase. The majority of research investigating synchrony’s effect on endorphin activity has focused on in-phase synchrony following vigorous activities. The only research to investigate the effects of anti-phase synchrony on endorphin activity found that anti-phase synchronized rowing did not produce the synchrony effect (Sullivan et al., 2014). Anti-phase synchrony, however, is counter-intuitive to the sport of rowing and may have interfered with the synchrony effect. This study investigated the effect of anti-phase synchrony on endorphin activity in a different task (i.e., drumming). University students (*n* = 30) were asked to drum solo and in in-phase and anti-phase pairs for 3 min. Pain threshold was assessed as an indirect indicator of endorphin activity prior to and following the task. Although the in-phase synchrony effect was not found, a repeated measures ANOVA found that there was a significant difference in pain threshold change among the three conditions [*F*(2,24) = 4.10, 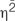 = 0.255, *p* < 0.05). *Post hoc t*-tests showed that the anti-phase condition had a significantly greater pain threshold change than both the solo and in-phase conditions at *p* < 0.05. This is the first time that anti-phase synchrony has been shown to produce the synchrony effect. Because anti-phase drumming may have required more attention between partners than in-phase synchrony, it may have affected self-other merging (Tarr et al., 2014). These results support Tarr et al.’s (2014) model that multiple mechanisms account for the effect of synchrony on pain threshold, and suggest that different characteristics of the activity may influence the synchrony effect.

## Introduction

Synchronized behavior has been linked to many positive social outcomes such as cooperation (Marsh et al., 2009; Valdesolo et al., 2010; Sullivan et al., 2015), affiliation (Hove and Risen, 2009), similarity, liking and connectedness (Valdesolo et al., 2010; Tarr et al., 2016) and performance (Davis et al., 2015) and is thought to be related to social bonding (Dunbar and Shultz, 2010). These outcomes may be due to the role of endorphins, which have been implicated in both pro-social behavior (Dunbar and Shultz, 2010) and synchronized actions (Cohen et al., 2010).

The underlying mechanisms influencing synchrony’s effect on endorphins and the potential link between synchrony, endorphins and social bonding is complex (Sullivan et al., 2015). Since endorphins do not cross the blood–brain barrier (Boecker et al., 2008), precise and accurate measurement can be challenging when indirect measures are used (Davis et al., 2015). Specifically, indirect measures may not be sensitive enough to reflect the precise neurological activity occurring in the brain. However, pain threshold has become a widely used and accepted indirect method of measuring neurological changes in endorphin activity (Jamner and Leigh, 1999; Dunbar et al., 2012).

The phenomenon known as the synchrony effect is a particularly fascinating mechanism within this context. Specifically, the synchrony effect refers to the finding that pre- to post-activity changes in pain threshold are significantly greater when activities are performed in synchronized groups compared to when they are performed alone (Cohen et al., 2010; Sullivan and Rickers, 2013; Sullivan et al., 2014) or unsynchronized (Tarr et al., 2015, 2016). The original study on the synchrony effect by Cohen et al. (2010) had twelve members of a rowing crew row for 45 min on ergometers in two counterbalanced conditions: rowing alone and synchronized in groups of six. Power output was equal in both conditions. Significant changes in pain threshold were found under both conditions from pre to post rowing. However, the changes in pain threshold were significantly greater when rowers rowed in synchrony compared to rowing alone. The authors speculated, that “synchronized activity somehow heightens opioidergic activity” (p. 108). Subsequent work has replicated this synchrony effect with rowing (Sullivan and Rickers, 2013; Sullivan et al., 2014).

More recent research using this synchrony effect protocol has supported the generalizability of the finding while specifying different factors that may affect it. Tarr et al. (2015) found that synchronized dance led to the synchrony effect. In their study, groups of three high school students danced for 10 min in one of four conditions differing in the level of exertion and synchrony. In the high exertion conditions, participants performed full body movements, whereas in the low exertion conditions, small hand gestures were synchronized while seated. In the synchronized conditions, all participants performed the same movements to the same music at the same time. In the partially synchronized conditions, participants performed different movements to the same music. The results revealed that both synchrony and exertion have positive *independent* effects on pain threshold as well as self reported measures of social bonding. This study is important in that it suggests that even activities requiring little physical exertion interact with the endogenous opioid system during synchronized movement, and possibly sheds light on a link between synchrony, endorphin activity and social bonding.

Tarr et al. (2016) also investigated the synchrony effect through three different dancing conditions. In groups of four, participants danced for 13 min wearing headphones that played music and instructions to a sequence of four basic dance moves that were previously learned. In the synchronized condition, participants heard the same instructions and the same music at the same time. In the partial synchrony condition, participants heard the same music at the same time but the sequence of dance moves varied so that none of the participants performed the same movements at the same time. In the asynchronyous condition, each participant performed a unique dance routine, and although the same musical tracks were heard, they were played at different times so that the timing of rhythm would be incongruent. Measures of pain threshold were taken before and after sessions. The researchers also measured self-reported social closeness, likability, connectedness, and personality similarity ratings.

The results showed that dancing in synchrony (i.e., same movements *and* same music) in small groups of strangers resulted in significantly greater changes in feelings of social closeness and pain threshold change from pre to post dance. Interestingly, partially synchronized dance (i.e., same music and off timed movements) led to an overall *decrease* in pain threshold change whereas asynchrony condition (i.e., different movements and off timed music) led to no change in pain threshold. The authors explained the results by suggesting that partially synchronized dance in this study may have resulted excessive distraction to participants, due to their awareness and knowledge of each other’s movements, and potentially caused a negative response.

One aspect of the synchrony effect that has received little attention is the pattern of synchrony. Synchrony has two dominant ‘patterns’; *in-phase* and *anti-phase* (Marsh et al., 2006; Richardson et al., 2007; Repp and Su, 2013; Sullivan et al., 2014). In-phase synchronized movements are conducted in perfect unison whereas anti-phase synchronized motions are conducted in alternating patterns of movement between two individuals. For example, if two people rock side by side in rocking chairs, rocking forward and backward in exact unison is termed in-phase synchrony. Comparatively, anti-phase synchrony is achieved when one individual is at a maximal forward position while the other is at a maximal backward position in exact unison (Richardson et al., 2007; Marsh et al., 2009). Despite its potential for unveiling salient links underlying the synchrony effect, anti-phase synchrony has received minor attention in the literature (Sullivan et al., 2014).

The only study to investigate the effects of anti-phase synchronized behavior on pain threshold was Sullivan et al. (2014). In this study, 22 experienced rowers completed three 30 min rowing sessions under solitary, in-phase and anti-phase conditions. In the in-phase condition, partners were in the fully extended and fully contracted positions of their strokes at the same time. In the anti-phase condition, rowers maintained synchrony by one rower being fully contracted when the other was fully extended (Sullivan et al., 2014). Pain threshold was measured 1 min before and after each trial, using the blood pressure cuff protocol from Cohen et al.’s (2010) original study. Interestingly, their results suggested that only in-phase synchrony, but not anti-phase synchrony, is capable of producing the synchrony effect, since significant changes in pain threshold were only seen in the in-phase condition. This would mean that something unique about in-phase synchrony affects endorphin levels. However, the nature of anti-phase movement in rowing may have affected the results. Recent research has found that anti-phase rowing can be effective in power production (de Brouwer et al., 2013; Cuijpers et al., 2015). However, the authors of theses studies note that in-phase rowing is much more typical and accepted by rowers during training and competition, and it has been suggested that synchrony may be particularly influential when participants are working toward shared goals and intentions (Tomasello et al., 2005). Potentially, this asynchronous rowing in Sullivan et al.’s (2014) study may have represented an unconventional activity may have interrupted the synchrony effect, and therefore, endorphin levels did not influence significant changes in pain threshold. This highlights the need for further investigation into the phases of synchrony.

To date, the literature in the area has shown that the effect of synchronized group behavior on pain threshold is a robust one. It has been replicated in different contexts and with different samples. However, there are still key characteristics about the task that may affect this phenomenon. Currently the most obvious one is the phase of synchrony. Although anti-phase synchrony is a common form of movement in groups, it has not been shown to support the synchrony effect. However, it is important to investigate the potential effect of anti-phase synchrony in different types of activities.

A scenario or activity where a conventional goal exists for synchronization in anti-phase and in-phase would provide a more accurate platform to investigate the effects of phase on the synchrony effect. Drumming is an activity conventional synchronization at both in-phase and anti-phase can exist depending on the desired sound and rhythm of the musical arrangement. Children as young as two are capable of synchronizing themselves with a drum beat (Kirschner and Tomasello, 2009) and drumming performance has also been found to affect changes in individual pain thresholds (i.e., endorphin levels; Dunbar et al., 2012) in line with other activities previously used to investigate the synchrony effect. Moreover, Weinstein et al. (2016) recently found that another musical activity, synchronized choir signing, can produce the synchrony effect. Additionally, since most research on the synchrony effect until recently (Tarr et al., 2015, 2016) has used highly vigorous activities, this study chose to employ a non-vigorous task.

The purpose of this study was to investigate the effect of anti-phase synchrony on endorphin activity in a more conventional anti-phase synchrony task. It was hypothesized that performing in-phase and anti-phase drumming would produce greater changes in pain threshold compared to drumming performed alone.

## Materials and Methods

### Participants

Thirty undergraduate students (17 male, 13 female) from a sport psychology course volunteered to participate in the study. They were granted 2% of toward their final grade for their participation. Ages ranged from 19 to 23 years old (*M* = 20.43, *SD* = 1.03). Inclusion criteria required that participants were not expert or highly experienced drummers. Based on the effect size of Cohen et al.’s (2010) study of 0.6, a β of 0.75 and α of 0.05, these studies would include sample sizes of 31 individuals for adequate power (Munro, 1997).

### Research Design

The current design was a repeated measures cross sectional design with pain threshold was the dependent variable. As in Cohen et al. (2010), there were conditions for both solo and in-phase synchrony. Furthermore, consistent with Sullivan et al. (2014), there was also an anti-phase synchrony condition. Both synchrony conditions were performed in pairs, but pairs comprised different individuals for each condition. Conditions were counterbalanced across individuals^1^. Each individual waited 2–3 days between each session.

### Procedure

Prior to data collection, all procedures were approved by the Research Ethics Board of the first author. The experiment was a repeated measures design. Therefore, participants were required to complete three counterbalanced drumming sessions; one solitary and two partnered (synchronized in-phase and anti-phase). Each session consisted of a 3-min, non-vigorous drumming task, and two measures of pain threshold.

All sessions were held in same room using the same drums and sticks, without an audience, aside from the researcher. Participants sat in a chair with a floor-standing tom drum in front of them at knee height, holding a stick in each hand, and would strike the drum one stick at a time to create and maintain a predetermined beat that was set by a metronome. In the solitary condition, participants sat with their drum in front of them, and faced another unoccupied chair and drum (used in the synchronized conditions). In both synchrony conditions, the partner occupied that chair.

In each condition, the researcher gave a brief drumming demonstration by synching with a metronome after explaining the session task (solo, in-phase or anti-phase). Specifically, a metronome sound set to the speed of 65 BPM played aloud and the researcher synched in-phase with the metronome in the solitary and in-phase conditions, and synched in anti-phase with the metronome in the anti-phase condition.

In the solitary condition, participants synched their strokes with the beat of the metronome. They would hit their right hand on the drum while holding their left hand up and away from the drum, and on the next beat (65 BPM later) hit their left hand on the drum while raising their right hand. The metronome was removed and they continued to drum for 3 min. In the in-phase condition, similarly, participants synchronized their left and right hand strokes with each other so that they would *both* hit the drum at the same time with their right hands, then their left hands, on pace with the metronome. The metronome was removed and they continued to drum for 3 min in in-phase synchrony. In the anti-phase condition, only one participant began to synchronize their left and right strokes to hit the drum on pace with the metronome as in the solitary condition. The second participant then entered the rhythm on the off beat, stroking their sticks on the drum at the opposite times of their partner. Specifically, after participant A stroked the drum with their right hand, participant B stroked the drum with right hand *before* participant A stroked the drum with their left hand. Participants continued this pattern and beat, creating a combined rhythm of 130 BPM, even though each participant was playing 65 BPM. This tempo has been used in previous synchrony research (Kirschner and Tomasello, 2009; Tarr et al., 2015); and is within the range of spontaneous tempo during actions such as tapping or drumming (Fitzpatrick et al., 1996; Provasi and Bobin-Bègue, 2003).

Pain threshold was used as an analysis of endorphin levels as per the protocol of Cohen et al. (2010) and Sullivan et al. (2014). Measurements were taken immediately before and after each drumming session. An algometer was used to put pressure on the first dorsal interosseous muscle of the non-dominant hand. The participant was instructed to let the researcher know when the pressure increased from *discomfort* to *pain* by saying “now.” At this point, the pressure was recorded as an indicator of pain threshold. Pre and post pain threshold measures were both taken by the same researcher for each participant.

## Results

**Table 1** shows the mean pain threshold values for before and after both sessions, as well as change scores.

**Table 1:** Means and standard deviations of pain threshold by condition.

Condition	Pre	Post
Solitary	54.33 (21.49)	52.10 (26.45)
In-phase	54.48 (26.18)	52.83 (25.79)
Anti-phase	53.56 (23.85)	57.04 (28.01)

*All values in mmHg.*

The pre-scores on the three conditions did not significantly differ [*F*(2,58) = 0.03, *p* > 0.05]. Pre to post-session change scores were used as dependent variables to test the hypotheses. Although there are some concerns with using raw change scores in such analyses, research has shown that when using reliable scores, particularly physiological measures, it is advisable to use raw change scores (Farrar et al., 2001; Dimitrov and Rumrill, 2003).

This data was checked for the assumptions of repeated measures. Inspection of boxplots of change score by condition revealed one significant outlier in the anti-phase condition. After this case was removed, change scores in all three conditions were normally distributed (Kolmogorov–Smirnoff tests *p* > 0.05). Mauchly’s test revealed that the assumption of sphericity was upheld (χ^2^ = 3.32, *p* > 0.05). Because both in-phase and anti-phase conditions occurred within pairs, it is possible that dependency between the scores existed within pairs nested within conditions. The intraclass correlation was calculated to assess the independence of the pain threshold change score as per the guidelines of Kenny et al. (1998). This resulted in an ICC of 0.106. The upper limit of ICC is 1.0 and scores closer to 0 indicate independent observations. These results suggest that the observations at the individual level were independent and that the individual, not the group, should be used as level of analysis (Kenny et al., 1998).

A repeated measures ANOVA found that there was a significant difference in pain threshold change among the three conditions [*F*(2,24) = 4.10, η^2^ = 0.255, *p* < 0.05]. Subsequent *post hoc* paired *t*-tests revealed that there was no significant difference between solo and in-phase conditions [*t*(25) = 0.14, *p* > 0.05], but anti-phase differed significantly from both solo [*t*(25) = -2.48, *p* < 0.05] and in-phase conditions [*t* (25)= -2.14, *p* < 0.05]. In both cases the average change in pain threshold was significantly higher in the anti-phase condition.

## Discussion

The current study was designed to see if the synchrony effect would be found under conditions of both in-phase and anti-phase synchrony, specifically in a task in which anti-phase interpersonal synchrony was a conventional activity. It was hypothesized that both in-phase and anti-phase drumming would produce significantly greater changes in pain threshold compared to solitary drumming.

The results partially supported the hypothesis, but in an interesting way that extends our understanding of this effect of synchrony on pain threshold. Specifically, it was found that although there was no significant difference in pain threshold change between control and in-phase conditions, there was a significant different between control and anti-phase synchrony. That is, anti-phase synchrony in this activity affected the synchrony effect whereas in-phase synchrony did not. This is the first study to suggest that the synchrony effect can be the result of anti-phase synchrony.

To date, the effect of in-phase synchrony on pain threshold appears to be fairly robust. Since Cohen et al.’s (2010) study, it has been replicated with rowing (Sullivan and Rickers, 2013; Sullivan et al., 2014), dance (Tarr et al., 2016), and singing (Weinstein et al., 2016). It has been found in group sizes from pairs (Sullivan et al., 2014) to hundreds (Weinstein et al., 2016), with both males and females, and strangers as well as teammates (Sullivan and Rickers, 2013). The only previous study to examine the potential role of anti-phase synchrony found that it did not induce any effect on pain threshold (Sullivan et al., 2014). However, that study used the activity of rowing, in which anti-phase synchrony is unconventional.

Although the current finding may appear to be inconsistent with previous research, recent developments in the conceptualization of synchrony, and the nature of the task in this study may explain these results. Tarr et al. (2014) suggested that endorphin release from musical conjoint activities could be the result of two distinct processes. One mechanism would be that these synchronized movements would induce activation of endogenous opioid system, perhaps through exertion, which is reflected in increased pain threshold. This is the pathway typically used to explain the synchrony effect in studies such as Cohen et al.’s (2010). The second mechanism, which is suggested to operate independently, or at least concurrently, with the first, is that synchronization can cause a blurring of the boundaries between self and others. This self-other merging can directly affect endorphin activation. According to Tarr et al. (2014),

when our own actions match those of another’s, it is possible that the intrinsic and extrinsic engagement of neural action- perception networks make it difficult to distinguish between self and perceived other, thus creating at least a transient bond between the two (p. 3).

This effect may be more powerful with music (Kuhn, 2002; Repp and Su, 2013), with meaningful goal directed movement (Tomasello et al., 2005), and in small groups (Tarr et al., 2014), consistent with the current design. There is substantial literature that shows that drumming (Kokal et al., 2011) as well as a wide variety of conjoint behaviors in general, has a positive effect on pro-social outcomes. For example, Morgan et al. (2017) recently published a meta analyses which concluded that interpersonal synchrony has small to medium effect sizes on social cognition and bonding outcomes. Similar reviews have drawn the same conclusions with respect to mimicry (Chartrand and Lakin, 2013) and interpersonal coordination (Marsh et al., 2009). Furthermore, it has been noted that these pro-social behaviors are influenced by the same endorphin activity that underlies this pain threshold effect (Dunbar and Shultz, 2010), suggesting that synchrony and similar activities affect cooperation through endorphin activity.

This self-other awareness may be a viable explanation for the change in pain threshold seen in the anti-phase condition, particularly considering it was not seen in the in-phase condition, or in the anti-phase condition of previous research (i.e., Sullivan et al., 2014). The anti-phase synchrony in the current drumming task may be a more engaging action than in-phase drumming synchrony. Whereas in-phase synchrony may have been simply mimicry, anti-phase was a goal-directed conjoint activity resulting in a significant group output, and inducing a significant effect on endorphin activity. This would explain the finding of a synchrony effect in anti-phase but not in-phase condition of the current study.

Furthermore, because the anti-phase rowing used by Sullivan et al. (2014) would not be a natural goal-oriented task (i.e., it is not typical for rowers to row in this fashion), this would not have activated the self-other mechanism for endorphin release as the current drumming task may have. Additionally, whereas the participants in Sullivan et al.’s (2014) study rowed side by side, participants faced each other in the current design, and Tarr et al. (2014) suggest that self-other merging depends on individuals being able to attend to each other. These factors would explain the difference in the anti-phase conditions between the two studies. By incorporating a goal-oriented task in a manner that explicitly included perception to action, the current study optimized self-other awareness and subsequent endorphin activity as seen in pain threshold changes.

There are potentially important practical implications of these results, particularly when considering the consequences of synchrony. Research has shown that synchrony can lead to a variety of social outcomes (e.g., cooperation, interpersonal attraction). Synchrony can induce these outcomes, but the most beneficial type of synchrony may depend on the typical type of movement in the activity. In activities that are typically performed in phase, such as rowing, we would predict that in-phase synchrony would be most likely to induce these outcomes. However, in activities that are also typically performed anti-phase synchrony, such as drumming, anti-phase synchrony would seem to be more efficacious. Other activities that may fit this pattern could include dancing, marching bands and boxing or footwork/shadowing drills in various sports.

We believe that the current results present a significant addition to the literature. However, there are some limitations that must be acknowledged. Although the sample size was adequate, a larger sample would have supported a more powerful design and may have helped to fully illuminate the effect of in-phase synchrony. There was also a potential confounding variable in the anti-phase condition. Because individual drummers were hitting the drum on their partner’s off beat, this resulted in a conjoint130 bpm, which is much closer to the preferred music bmp (120; McKinney and Moelants, 2006) than the 65 bpm in the in-phase condition. It is possible that the significant difference between conditions may be due, not to anti-phase synchrony, but the bpm in the anti-phase condition. A secondary aspect of the anti-phase condition was that this activity has a leader-follower dynamic in which the conjoint group task is initiated by one persona and then followed by the second. However, this dynamic would not sustain as after the beat is established it becomes very cyclical and therefore hard to determine who is following whom. Finally, the participants and the task may also limit the external validity of the findings. The sample comprised novice drummers and a relatively basic drumming task. Would the same results be seen with experts and a more complicated task?

Future research should do more to address these issues. Primarily, we would suggest that it is time to include a manipulation check and expand on the outcome measures of the typical synchrony effect protocol, as established by Cohen et al. (2010). Typically in these studies synchrony is manipulated as either present or absent, and it is assumed, because the participants are typically highly trained or skilled, that they are completely and uniformly synchronized. However, we would suggest that it would be a valuable addition to the literature to actually assess the degree of synchrony among members in the synchrony condition. Other potential covariates such as movement rate would also be a valuable addition to this protocol. Furthermore, we suggest that the generalizability of these results should be further examined. If the meaningful nature of the task is an important factor in the synchrony effect, what are the limitations of a meaningful task? This is particularly important as recent research is examining the effect of synchrony with real world tasks (e.g., Tarr et al., 2016; Weinstein et al., 2016). Is it possible that different phases on synchrony might be meaningful in real life tasks? For example, drum groups or marching bands may exercise movements that are synchronized but neither in-phase nor anti-phase. Also, the role of competition deserves research. If in-phase synchrony is a relevant goal-oriented activity in rowing, is it more goal-oriented in competition than in training? Future clarification of these and other issues will do much to further our understanding of the fascinating phenomenon of the synchrony effect.

## Author Contributions

PS was responsible for the overall design of this study and was primarily responsible for data analysis and writing. MB was involved in all aspects of the study. She was primarily responsible for REB approval and data collection, and was actively involved in writing all components of the manuscript.

## Ethics Statement

This study was carried out in accordance with the recommendations of the Research Ethics Board of Brock University with written informed consent from all subjects. All subjects gave written informed consent in accordance with the Declaration of Helsinki. The protocol was approved by Brock University’s Research Ethics Board.

## Conflict of Interest Statement

The authors declare that the research was conducted in the absence of any commercial or financial relationships that could be construed as a potential conflict of interest.

## Footnotes

1 Two individuals’ schedule interfered with counterbalancing.
